# Prognostic relevance and validation of ARPC1A in the progression of low-grade glioma

**DOI:** 10.18632/aging.205952

**Published:** 2024-06-19

**Authors:** Jingyuan Dai, Jiahui Gao, Hongchao Dong

**Affiliations:** 1 School of Computer Science and Information Systems, Northwest Missouri State University, Missouri, MO 64468, USA; 2 Cangzhou Hospital of Integrated Traditional Chinese and Western Medicine, Hebei, China; 3 Shaanxi Second People’s Hospital, Shaanxi, China

**Keywords:** low-grade glioma, mitochondria-genes, ARPC1A, MAPKinases, nomogram, prognosis, TCGA, CGGA

## Abstract

Low-grade glioma (LGG) is a grade II-III glioma accompanied by distinct clinical and molecular characteristics and the studies related to its prognosis are still unclear. The objective of this study is to explore the involvement of mitochondrial-related genes SLBP, COMMD7, LSM4, TOMM34, RPP40, FKBP1A, ARPC1A, and TBCA for the prognosis of LGG. We detected differences in the expression of some of the genes by analyzing the bioinformatics dataset and combining it with RT-PCR experiments. Subsequently, a nomogram was constructed and validated for the clinical relevance of risk factors such as age, WHO grade, IDH mutation status, Ch.1p19q co-deletion status, and high and low expression of ARPC1A to predict the 1-, 3-, 5-year overall survival and prognostic relevance of ARPC1A. Gene set enrichment analysis was performed for the relevant datasets pertinent to the expression of ARPC1A to elucidate the cancer-promoting pathways involved in the LGG through KEGG and GO analysis. Transfection assays, CCK-8 assays, and flow cytometry were used to determine the proliferation rate, and apoptosis rate of the HS683 and SW1783 cell lines respectively. Western blotting was used to examine the involvement of the cancer-promoting activity of ARPC1A through MAPK signaling. In this study, the prognostic value of ARPC1A in LGG was found by bioinformatics analysis combined with experimental approach analysis and may be a significant independent risk factor. ARPC1A fosters a higher LGG proliferation rate that may control the MAP kinase signaling and could be a prominent biomarker for LGG. Future studies are warranted to explore its clinical implications.

## INTRODUCTION

Gliomas are aggressive primary tumors confined to the central nervous system and their incidence rates have been increasing and account for 40% to 50% of the total incidence of intracranial tumors. These cancers are highly malignancy and cause a decline in the overall survival of patients worldwide due to the lack of suitable early prognostic strategies [1–4]. Mainly, the LGG is a grade II-III glioma composed of different clinical and molecular characteristics [5]. A growing number of WHO low-grade gliomas are incidentally observed but some of the patients do not manifest LGG-related symptoms and these incidental LGGs are uncommon and account for 3.8–10.4% of all the LGGs [6–13]. Clinical treatment modalities for gliomas include surgery, radiotherapy and chemotherapy and their implications have failed to provide effective clinical outcomes due to poor clinical prognosis [14, 15]. Surgical treatment is one of the primary therapeutic modalities for patients who do not manifest tumor-related symptoms [16–21]. Survival-related factors could help determine surgical decisions. A few retrospective reports described that the overall survival of patients diagnosed with ‘incidental LGG’ is satisfactory when compared to the patients with symptomatic LGG [8, 9]. LGG is exemplified by the progressive growth and the asymptomatic incidental LGG to symptomatic LGG may last for longer years [10, 16–19, 22, 23].

Mitochondria plays a crucial role in various disease development, including cancers [24–30]. When the division process of mitochondria is accelerated, it will promote cancer proliferation and invasion, and even metastasis [31]. The mitochondrial autophagic process, when augmented, likewise promotes tumor survival [32]. For example, FAM72A promotes glioma progression by participating in the induction of mitochondrial division and phagocytosis in gliomas [33]. In addition, abnormalities in mitochondrial regulatory pathways can also affect alterations in gene expression profiles, which in turn can lead to tumor progression and possibly even tumor immune escape [34]. Glioma cells are significantly dependent on oxidative phosphorylation to produce ATP inside the mitochondria whereas the glioma cells specifically prefer aerobic glycolysis that can induce OXPHOS without glucose [35, 36]. Hence, the metabolic reprogramming can be observed through the mitochondria in tumor cells depending on the microenvironment of the tumor. Mitochondrial-related genes have significant relation to the prognosis of glioma patients which requires substantial studies [37, 38]. Unlike glioblastoma, LGG mainly develops in younger individuals [39]. According to the TCGA database, more than 90% of LGG patients are below 60 years of age. Grade, age, and IDH mutational status can affect overall survival. For instance, the pathological age can influence tumor incidence and overall survival of LGG patients. Past studies described the prognostic relevance of EMP3 [5, 40–43] and IGFBP2 [44–46] in glioma. TIMP1 and SERPINE1 genes are significantly upregulated in the LGG patients of older age [5].

However, the prognostic relevance of various mitochondria-related genes in LGG and their involvement in cancer promotion require several studies. In this study, we screened several mitochondria-related genes for their prognostic relevance for LGG and subsequently elucidated the importance of ARPC1A to predict the prognosis of LGG.

## MATERIALS AND METHODS

### Data source and processing

High-throughput RNA sequencing expression profiles including mRNA and their correlated clinical data pertinent to the patients with LGG were downloaded from The Cancer Genome Atlas (TCGA: UCSC Xena- http://xena.ucsc.edu) and Chinese Glioma Genome Atlas (CGGA). We collected gene expression data from 182 LGG patients from a dataset of 325 glioma patients in the CGGA database [47], as well as gene expression data from 20 normal brain tissues for subsequent analysis. We first obtained the mitophagy gene set (29 marker genes are derived from the gene set provided by the GSEA official website https://www.gsea-msigdb.org/; Supplementary Table 1), and obtained the corresponding scores through the GSVA algorithm (Supplementary Table 2). “GSEABase”, “clusterProfiler”, “GSEA” and “GSVA” packages were used for gene set scoring as well as enrichment analysis. We performed Pearson correlation analysis in the LGG cohort of TCGA and CGGA. The screening criteria were correlation r > 0.4 or r < −0.4 (*p* < 0.001). There were 490 eligible genes in TCGA and 490 eligible genes in CGGA. There are 636 genes, and after the intersection, there were 161 overlapping genes (Supplementary Tables 1–3). We have not received any third-party support in conducting this research, analyzing the data, or preparing the manuscript for submission. Furthermore, “enrichplot” and “Clusterprofiler” packages were used to analyze the identification of GO-enriched terms and KEGG pathways.

### Bioinformatics analysis

Since there are fewer data on normal brain tissues adjacent to the cancer of gliomas in the TCGA database, we obtained sequencing data of 207 normal brain tissues from the Genotype-Tissue Expression (GTEx: https://www.gtexportal.org/) database for differential analysis. The normalize between arrays function in our limma package merges the normal brain tissue expression data from the GTEx database with the LGG expression data from TCGA after correction. Datasets from TCGA were typically used for the expression differences of several genes such as *SLBP, COMMD7, LSM4, TOMM34, RPP40, FKBP1A, ARPC1A*, and *TBCA*. The comparative expression differences were performed between cancerous tissues and noncancerous tissues. Subsequently, the overall survival and clinical correlations were executed. R-statistical computing includes “survival” (Kaplan-Meier curve survival) and “survminer” packages have been implicated to describe survival and Cox analysis; “beeswarm” used for clinical correlation analysis subsequently determined the prognostic value of these mitophagy-related genes.

### Gene enrichment analysis (KEGG and GO)

Prognostic value and expression differences of ARPC1A were determined by analyzing the corresponding GSEA datasets obtained from TCGA and CGGA to predict its cancer-promoting mechanism in LGG. In this analysis, the biological process pertinent to ARPC1A expression was analyzed through gene ontology and the KEGG pathway. The rich-GO containing R function was implicated for GO analysis whereas the enrich-KEGG pathways R function was used for KEGG pathway analysis. Here, the *P*-values were used to describe the related functions or pathways. (Critical value: 0.05 was recommended).

### Analysis of immune cell infiltration

In this study, the scores of 22 immune cell infiltrations in the TCGA-LGG dataset were evaluated using the CIBERSORT (Cell-type Identification by Estimating Relative Subsets of RNA Transcripts). The high and low expression of ARPC1A and immune cell infiltration were analyzed by difference analysis and correlation analysis.

### Nomogram analysis: construction and evaluation

Clinical information pertinent to the LGG patients from the TCGA database delineating the expression profiles of ARPC1A was obtained. We obtained clinical information that included age, gender, WHO grade, IDH mutation status, Ch.1p19q co-deletion status. First, we analyzed the correlation between high and low expression of ARPC1A in different clinical information including gender, WHO grade, IDH mutation status, Ch.1p19q co-deletion status. Univariate and multivariate Cox regression models were typically employed to screen and analyze these variables. A total of five factors were obtained that have significance including on the prognosis of LGG patients through ARCP1A expression. A nomogram was constructed and C-index, ROC analysis was used to evaluate its consistency.

### Cell cultures

Low-grade glioma cell lines such as HS683 and SW1783 were obtained from Land Biotechnology, (Guangzhou, China). The cell lines were cultured in DMEM media with 10% FBS and incubated at 37°C in 5% CO_2_. Later, they were subjected to transfection assays for knockout of ARPC1A to examine its expression profiles in these two cell lines using the following procedures.

### iRNA knockout assays

Suitable selection of SiRNA and plasmid construction for knocking out the ARPC1A was performed as per the protocols provided by Wang et al. [48]. The specific SiRNA and overexpression plasmids screened for this study were prepared by Land Biotechnology (Guangzhou, China). Lipofectamine 2000 (Invitrogen, USA) for transfecting siRNA and overexpression plasmid into the cell lines was executed according to the manufacturer’s procedures.

### Cell counting kit-8 (CCK-8)

Approximately 7.5 × 10^3^ HS683/SW1783 cells per well were seeded into 96-well plants and cultured in a CO_2_ incubator at 37°C. Parallelly, the control groups were also cultured for 24, 36, 48, 72, and 96 hours respectively. CCK-8 reagent of 10 microliters was added to each well and the reaction was allowed to react for 90 minutes. Later, the plates were subjected to full-wavelength microplate reading and the absorbance was measured at 450 nm.

### Apoptosis and flow cytometry

Annexin V-FITC apoptosis detection kit was procured from Beyotime, China, and the apoptosis rate was examined as per the manufacturer’s procedures. A total of 1 × 10^5^ cells were taken and the apoptosis rate was determined by adding 500 microliters of binding buffer and 5 microliters of Annexin V-FITC, and 5 microliters of propidium iodide in a flow tube subsequently, they were incubated at room temperature for 10 minutes and subjected to flow cytometry.

### RT-PCR studies

To assess the relative expression of mRNA, we employed the SYBR green method in conjunction with the Applied Biosystems 7500 RT-PCR system (USA). The PCR reactions were conducted using a 25 μl SYBR green master mix and a suitable primer at a concentration of 10 pM. The PCR protocol involved an initial denaturation step at 95ºC for two minutes, followed by 40 cycles of denaturation at 94ºC for 15 seconds, annealing at 60ºC for 1 minute, and extension at 72ºC for 15 seconds. RNA was isolated from the LGG samples and confirmed by the spectrophotometry with OD260/OD280 ratio which was found to be 1.9 to 2.1 indicating its good purity. Gene expression levels were determined based on Ct values and then normalized using the 2^−ΔΔCt^ method relative to an endogenous control. The resulting data were presented as fold changes in the expression levels of target genes. To ensure reproducibility, all experiments were performed in triplicates, and the details of the primers used can be found in Table 1.

**Table 1:** Primer sequences used for the RT-PCR to elucidate the expression profiles of mitochondrial autophagy-related genes.

**Gene**	**Primer**	**Sequence**
SLBP	Forward primer	5′-GACCCGAGAGCTTTACCACTC-3′
	Reverse primer	5′-GGCACAGTAGACATAGACTCCT-3′
COMMD7	Forward primer	5′-TCCAGGCGGATTTCATAACTCT-3′
	Reverse primer	5′-CACTTTCTCCAATTCGCTGCT-3′
LSM4	Forward primer	5′-GGGAGACGTACAATGGACACC-3′
	Reverse primer	5′-ACGTGCAGATGACTTCTCGC-3′
TOMM34	Forward primer	5′-TGCATCAAAGATTGCACTTCAGC-3′
	Reverse primer	5′-GCAGCACAGTCTTATAGTCAACA-3′
RPP40	Forward primer	5′-TTTGGCTTGGCATAAAACAGGT-3′
	Reverse primer	5′-CTCAACGTGCTCAGTGCTACT-3′
FKBP1A	Forward primer	5′-CTCCAGATTATGCCTATGGTGC-3′
	Reverse primer	5′-AGCTCCACATCGAAGACGAGA-3′
ARPC1A	Forward primer	5′-ATTGCCCTCAGTCCCAATAATCA-3′
	Reverse primer	5′-CAAGTGACAATGCGGTCGC-3′
TBCA	Forward primer	5′-CCTCGCGTGAGACAGATCAAG-3′
	Reverse primer	5′-CAAATATGCGGCTTCCAACCT-3′
β-Actin	Forward primer	5′-AGTGGGGTGGCTTTTAGGATG-3′
	Reverse primer	5′-ACAGCCTATGTCTGCAAACTCCACT-3′

### Western blotting

Protein lysates from knockdown cells and control groups were prepared using a centrifugation procedure, subsequently the lysates were treated with RIPA cocktail buffer at 4°C. SDS-PAGE was employed to separate proteins, which were then transferred onto a nitrocellulose membrane. Subsequently, the membranes were blocked at room temperature with skimmed (nonfat) milk in Tris-buffered saline containing 0.05% Tween 20 (TBST) for one hour. The blots were then probed with primary antibodies, including ARPC1A, and MAPKinase pathway proteins (ERK-1/2 and p-ERK1/2), Vimentin and GAPDH (a positive control). This incubation took place overnight at 4°C with gentle shaking. Following the primary antibody incubation, the blots were washed three times in TBST and then incubated with a secondary antibody. ECL (1:1 ratio of reagent 1 and reagent 2) was prepared and the blots were treated with ECL and consequently detected the banding pattern in Chemi-UV tech.

### Data analysis

Statistical analyses were performed using the R software 3.5.0. The *p*-values of less than 0.05 were considered as statistically significant. The overall survival was deciphered using Kaplan-Meier analysis to determine the prognostic relevance of ARPC1A expression and the group comparisons were performed with the aid of a log-rank test. In addition, the data were represented in the form of mean ± SD. Student’s *t*-test and one-way ANOVA were used to elucidate the significant differences among the groups. Univariate or multivariate Cox regression analysis was used to examine the prognostic relevance using R-statistical computing version 3.6.2.

## RESULTS

### Screening mitophagy-related genes and their prognostic relevance

**Figure 1:**
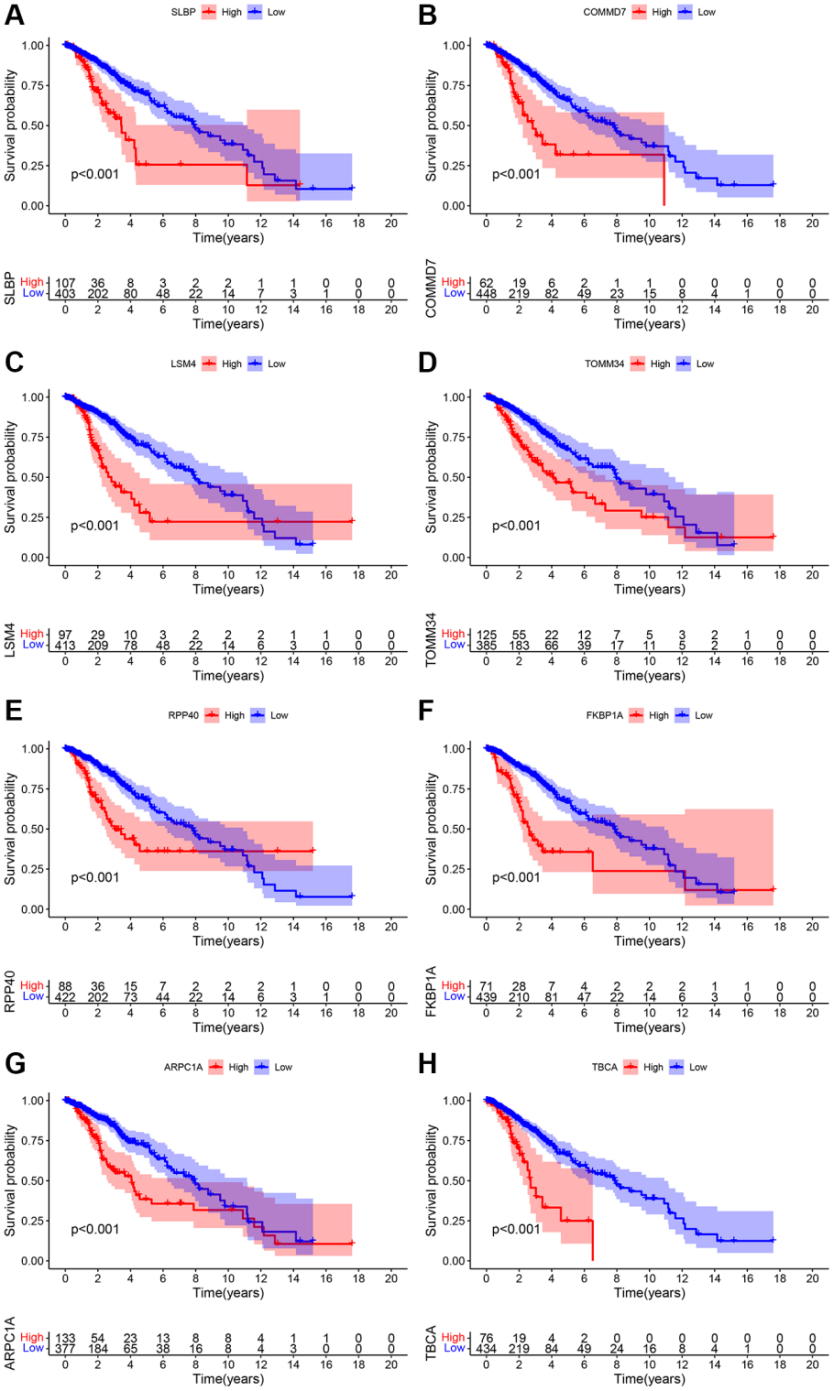
(**A**–**H**) Overall survival analysis pertinent to the mitochondrial-related eight genes include SLBP, COMMD7, LSM4, TOMM34, RPP40, FKBP1A, ARPC1A, and TBCA and their expression data and prognostic information of the TCGA database.

Pearson correlation analysis was used for the analysis of the LGG cohort obtained from the TCGA and CGGA database. Screening criteria were correlation r > 0.4 or r <−0.4 (*p* < 0.001). A total of 490 eligible genes in TCGA and 490 eligible genes in CGGA were screened. Later, a total of 636 genes, and after the intersection, there were 161 overlapping genes (Supplementary Table 3) were screened. Next, KEGG enrichment analysis was performed for these 161 genes, and the results suggested that this group of genes may be involved in the metabolism of substances such as amino acids, ribosomes and cholesterol (Supplementary Table 4). Overall survival analysis and Cox regression analysis were used to determine the prognostic value of these mitophagy-related genes. The results of the two data sets were *p* < 0.05, and the risk ratio HR >1. Finally, eight genes such as SLBP, COMMD7, LSM4, TOMM34, RPP40, FKBP1A, ARPC1A, and TBCA, and their co-relation on the patient’s overall survival was obtained (Figure 1, Supplementary Figure 1 and Table 2).

**Table 2:** Prognostic analysis of mitochondrial autophagy-related genes.

**Gene list**	**TCGA Dataset**			**CGGA Dataset**		
	**KM *p*-value**	**HR**	**Cox *p*-value**	**KM *p*-value**	**HR**	**Cox *p*-value**
SLBP	9.13e-03	1.043 (1.007–1.081)	1.90e-02	4.62e-03	3.144 (1.785–5.536)	7.27e-05
COMMD7	8.32e-04	1.068 (1.036–1.101)	2.18e-05	4.33e-03	3.449 (1.904–6.249)	4.42e-05
LSM4	9.24e-05	1.016 (1.011–1.022)	1.99e-08	4.48e-02	1.935 (1.404–2.668)	5.59e-05
TOMM34	1.26e-02	1.041 (1.004–1.078)	2.73e-02	1.87e-03	2.042 (1.373–3.036)	4.20e-04
RPP40	1.11e-02	1.175 (1.062–1.301)	1.81e-03	1.68e-03	2.144 (1.390–3.307)	5.59e-04
FKBP1A	3.40e-03	1.013 (1.008–1.017)	5.64e-08	1.69e-03	2.938 (1.903–4.535)	1.14e-06
ARPC1A	2.91e-06	1.017 (1.008–1.027)	1.46e-04	4.07e-03	2.804 (1.614–4.871)	2.55e-04
TBCA	2.57e-03	1.009 (1.005–1.014)	6.57e-05	1.71e-02	2.431 (1.483–3.98	4.27e-04

### Differential genes and verification by PCR experiments

**Figure 2:**
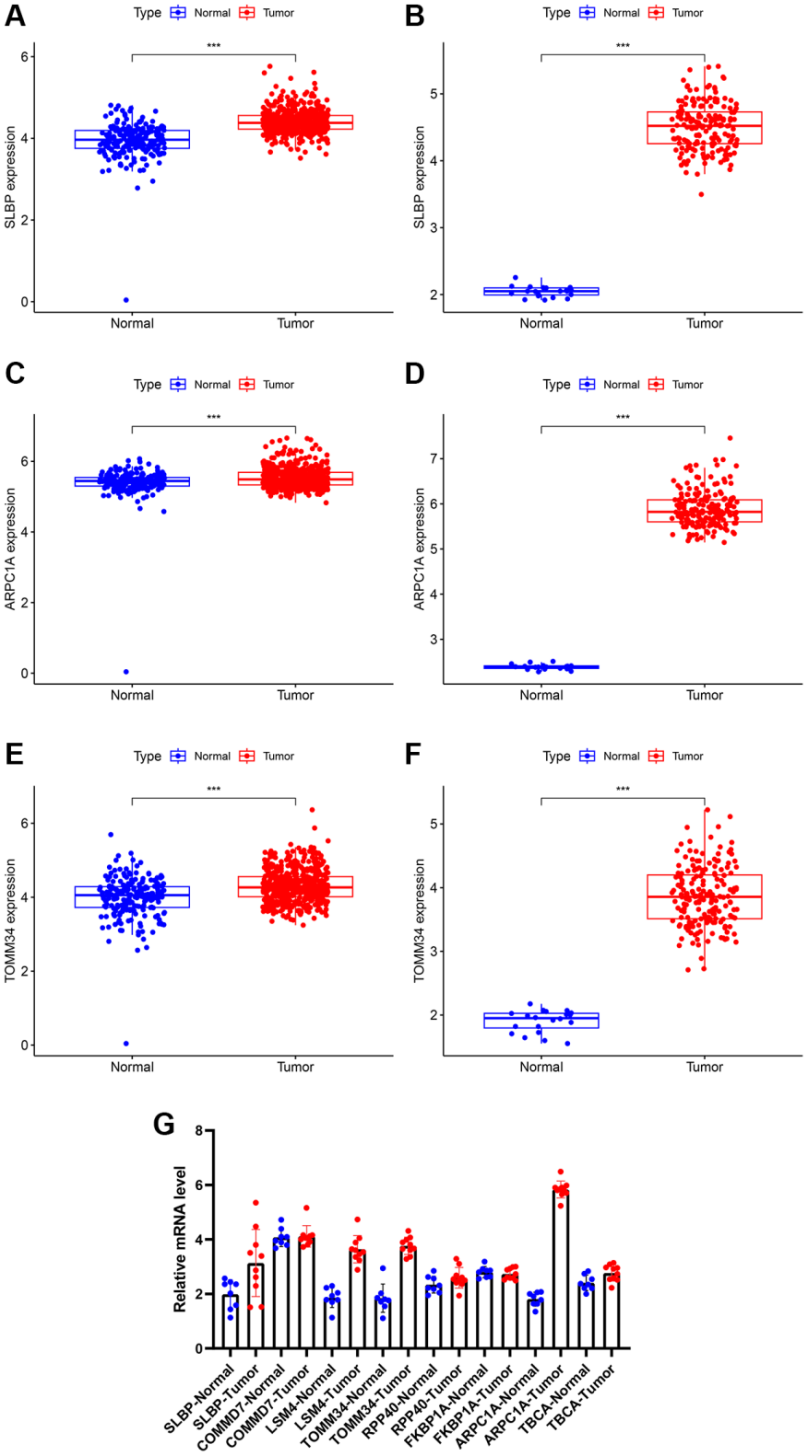
(**A**, **B**) Differential expression of analysis of SLBP based on TCGA and CGGA expression. (**C**, **D**) Differential expression of analysis of ARPC1A based on TCGA and CGGA expression. (**E**, **F**) Differential expression of analysis of TOMM34 based on TCGA and CGGA expression. (**G**) A higher mRNA expression of ARPC1A was evident in LGG tissues than normal tissue as indicated by the RT-PCR experiments.

Differential analysis through the expression matrix pertinent to mitochondria-related genes in the public database was performed and verified through RT-PCR experiments on clinical specimens (Table 2). Due to the lack of normal control sample data in the TCGA related to low-grade glioma, we combined TCGA and GTEx for differential analysis; subsequently, the normal brain tissue data was obtained from CGGA for differential analysis. Expression of the mitochondria-related genes including SLBP, ARPC1A, and TOMM34 (TCGA database) were significantly higher in cancer tissues than the normal tissues (Figure 2A, 2C, 2E). Expression of TBCA, COMMD7, LSM4, and FKBP1A were significantly lower in the cancer tissues than the normal tissues. However, there was no significant difference observed in the expression of RPP40 (Supplementary Figure 2A, 2C, 2E, 2G, 2I). In the case of CGGA, the expression of SLBP, ARPC1A, and TOMM34 were greatly differed between LGG cancerous tissues and adjacent normal tissue (Figure 2B, 2D, 2F) and (Supplementary Figure 2B, 2D, 2F, 2H, 2J). The expression of these mitochondria-related genes was examined through RT-PCR studies. The expression difference of ARPC1A was significantly higher when compared to all other genes and also higher than the adjacent genes and its role in the progression was determined through the following studies (Figure 2G).

### Nomogram significance for ARPC1A-prognostic relevance

**Figure 3:**
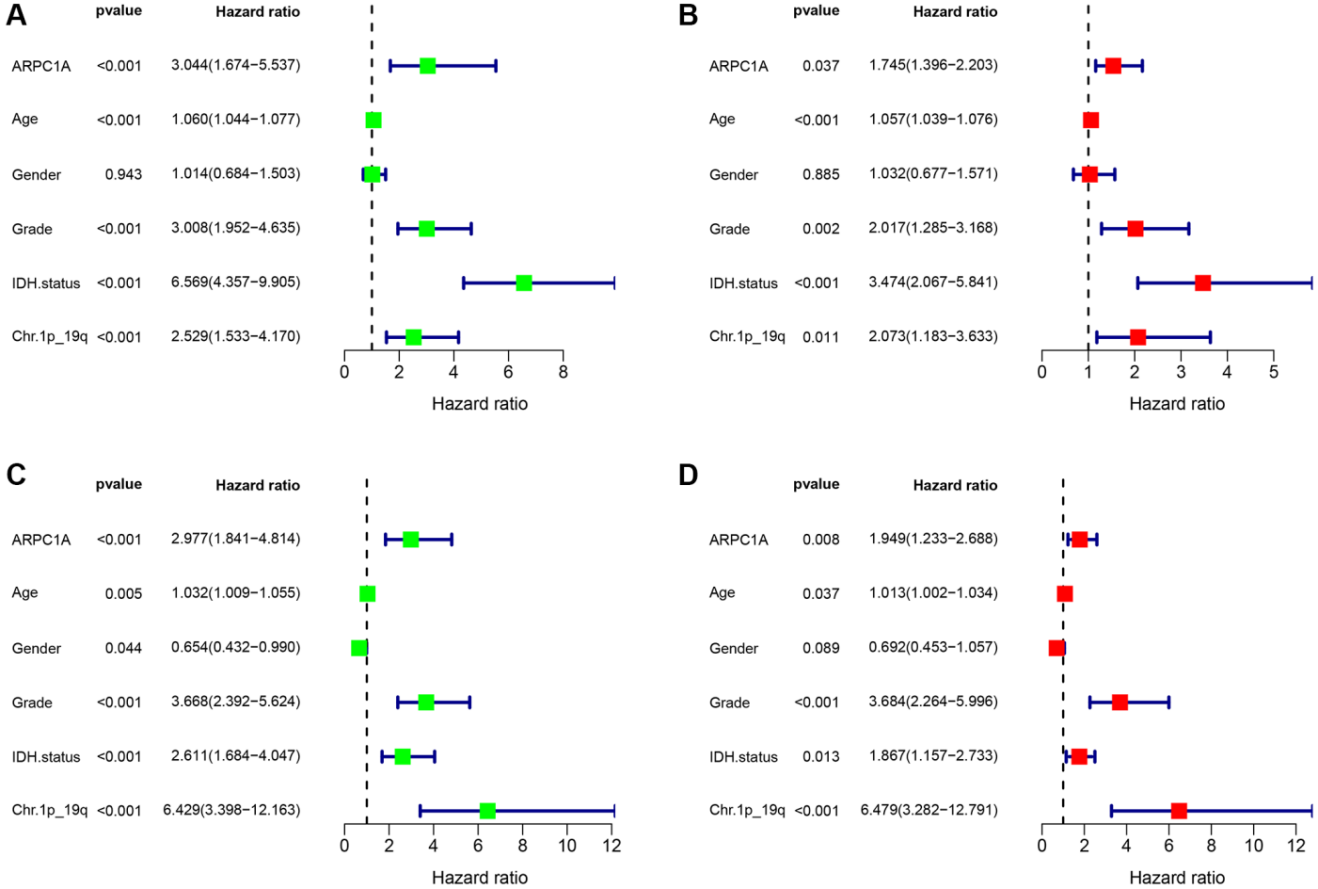
(**A**, **B**) Single-factor and multi-factor Cox regression analysis described the risk factors such as age, grade, expression profiles of ARPC1A, IDH mutation status, and Ch.1p_19q co-deletion status based on the prognostic information in the TCGA database; (**C**, **D**) Single-factor and multi-factor Cox regression analysis pertinent to risk factor such as age, grade, expression profiles of ARPC1A, IDH mutation status, and Ch.1p_19q co-deletion based on the prognostic information in the CGGA database.

**Figure 4:**
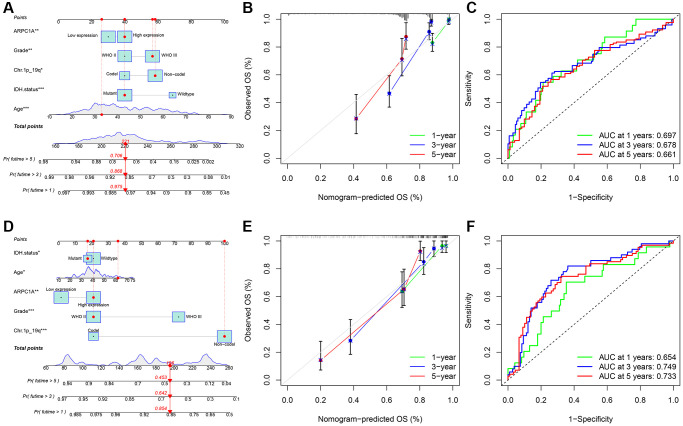
(**A**–**C**) TCGA: the nomogram model was constructed and validated through overall survival analysis based on the expression and prognostic information in the TCGA database; ROC analysis of ARPC1A in patients with LGG associated with AUC (area under the curve) validation based on TGGA. (**D**–**F**) CGGA: the nomogram model was constructed based on the expression and prognostic information and verified through a calibration curve; ROC analysis of ARPC1A in patients with LGG associated with AUC (area under the curve) validation based on CGGA.

We analyzed common clinical risk factors for glioma, including age, gender, WHO grade, IDH mutation status, 1p19q co-deletion status, and high and low expression of ARPC1A. The results of the correlation analysis suggesting the expression of ARPC1A with WHO grade and IDH mutation status were validated in both the TCGA and CGGA databases, suggesting that the expression of the gene was significantly higher with increasing grade and inside wild-type patients with IDH status (Supplementary Figure 3C, 3D, 3E, 3F). While gender and 1p19q co-deletion status were only validated in one database (Supplementary Figure 3A, 3B, 3G, 3H). Depending the data obtained from TCGA, Cox regression analysis described that age (*p* < 0.001, *p* < 0.001), WHO grade (*p* < 0.001, 0.002), IDH mutation status (*p* < 0.001, *p* < 0.001), 1p19q co-deletion status (*p* < 0.001, 0.011) and high and low expression of ARPC1A (*p* < 0.001, 0.037) were all related to poor prognosis (Figure 3A, 3B). Depending on the data obtained from CGGA, Cox regression analysis described that age (0.005, 0.037), WHO grade (*p* < 0.001, *p* < 0.001), IDH mutation status (*p* < 0.001, 0.013), Ch.1p_19q co-deletion status (*p* < 0.001, *p* < 0.001) and high and low expression of ARPC1A (*p* < 0.001, 0.008) were all related to poor prognosis (Figure 3C, 3D). Hence, a nomogram through these factors was constructed and the calibration curve was verified (Figure 4A, 4D). The nomogram model including the high and low expression of ARPC1A can bestow better prediction of 1-, 3-, 5-year survival rates pertinent to the prognosis of LGG patients; analysis of receiver operating characteristic (ROC) curve reported that ARPC1A is a significant predictor of 1-year, 3-year, and 5-year survival (Figure 4B, 4C, 4E, 4F).

### GO and KEGG enrichment analysis of mRNAs

**Figure 5:**
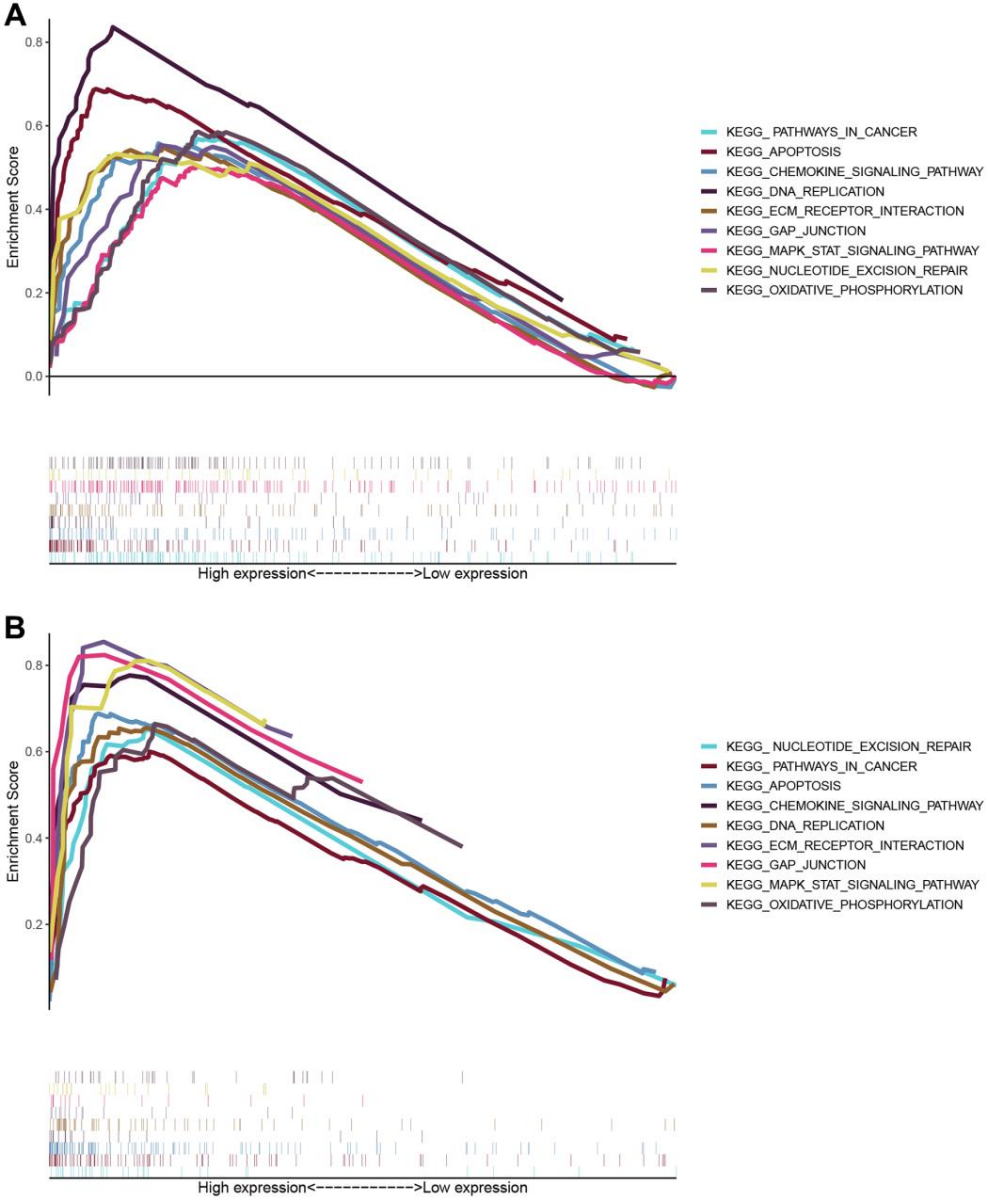
**The enrichment scores for biological processes in KEGG and GO enrichment.** The enriched biological processes and signaling pathways pertinent to protein-coding genes were correlated with the prognostic relevance of the ARPC1A signature. (**A**) KEGG enrichment analysis based on TCGA database; (**B**) GSEA results of KEGG pathway based on CGGA database.

Gene ontology (GO) and KEGG enrichment analysis using GSEA datasets were carried out to delineate the significant implications of mRNAs. Enrichment analysis showed that mRNAs were highly enriched in regulating signaling pathways in cancers such as KEGG_apoptosis, KEGG_DNA replication, and gap junction and oxidative phosphorylation pathways, including MAPK signaling pathways and chemokine signaling pathways in KEGG (Figure 5). Mainly, the expression differences of ARPC1A in its involvement in the cancer promotion in LGG specifically from the TCGA and CGGA datasets. It may affect the DNA replication process, thereby affecting tumor proliferation and apoptosis. It also suggests that tumor progression is affected by regulating signaling pathways in cancer, including MAPK signaling pathways and chemokine signaling pathways (Table 3); from the results. It can be concluded that the multiple mechanisms of ARPC1A regulate tumors in LGG. Next, ARPC1A is involved in the proliferation of cancer cells, and its involvement in apoptosis was also described through knockout experiments. On this basis, we performed genome-wide correlation analyses in the TCGA and CGGA databases, respectively, to identify significant genes with correlations r > 0.4 or r < −0.4 with ARPC1A. A total of 558 genes were matched in the TCGA dataset, while 931 genes were matched in the CGGA database, and 282 genes were obtained after the fetch intersection operation (Supplementary Table 5). KEGG enrichment analysis of these 282 genes resulted in the identification of genes that may affect cell cycle, DNA replication, mismatch repair, and nucleotide metabolism, among other functions (Supplementary Table 6).

**Table 3:** Normalized enrichment scores in KEGG pathway and GO analyses.

**Description**	**Normalized enrichment score (NES)**	***P*-value**
KEGG_PPAR_SIGNALING_PATHWAY	−1.846	<0.001
KEGG_P53_SIGNALING_PATHWAY	2.432	0.001
KEGG_MISMATCH_REPAIR	2.047	0.002
KEGG_DNA_REPLICATION	2.374	<0.001
KEGG_CELL_CYCLE	2.687	<0.001
GO_DNA_RECOMBINATION	2.455	<0.001
GO_DNA_REPAIR	2.415	<0.001
GO_DNA_REPLICATION	2.846	<0.001
GO_CELL_CYCLE_CHECKPOINT	2.789	<0.001

### ARPC1A and immune cell infiltration

Based on the results of immune infiltration analysis we found an association between ARPC1A expression in LGG and infiltration of T cells CD8, T cells CD4 memory resting, T cells follicular helper, Monocytes, Macrophages M0, Macrophages M1, Mast cells activated and Eosinophils (Supplementary Figure 4A–4I). However, the results of the correlation analysis demonstrated between −0.2 and 0.3, failing to reach the boundaries of the existence of a correlation greater than 0.4 or less than −0.4. Previous results suggest that ARPC1A has less effect on immune cell infiltration in LGG.

### Cancer-promoting mechanism of ARPC1A

**Figure 6:**
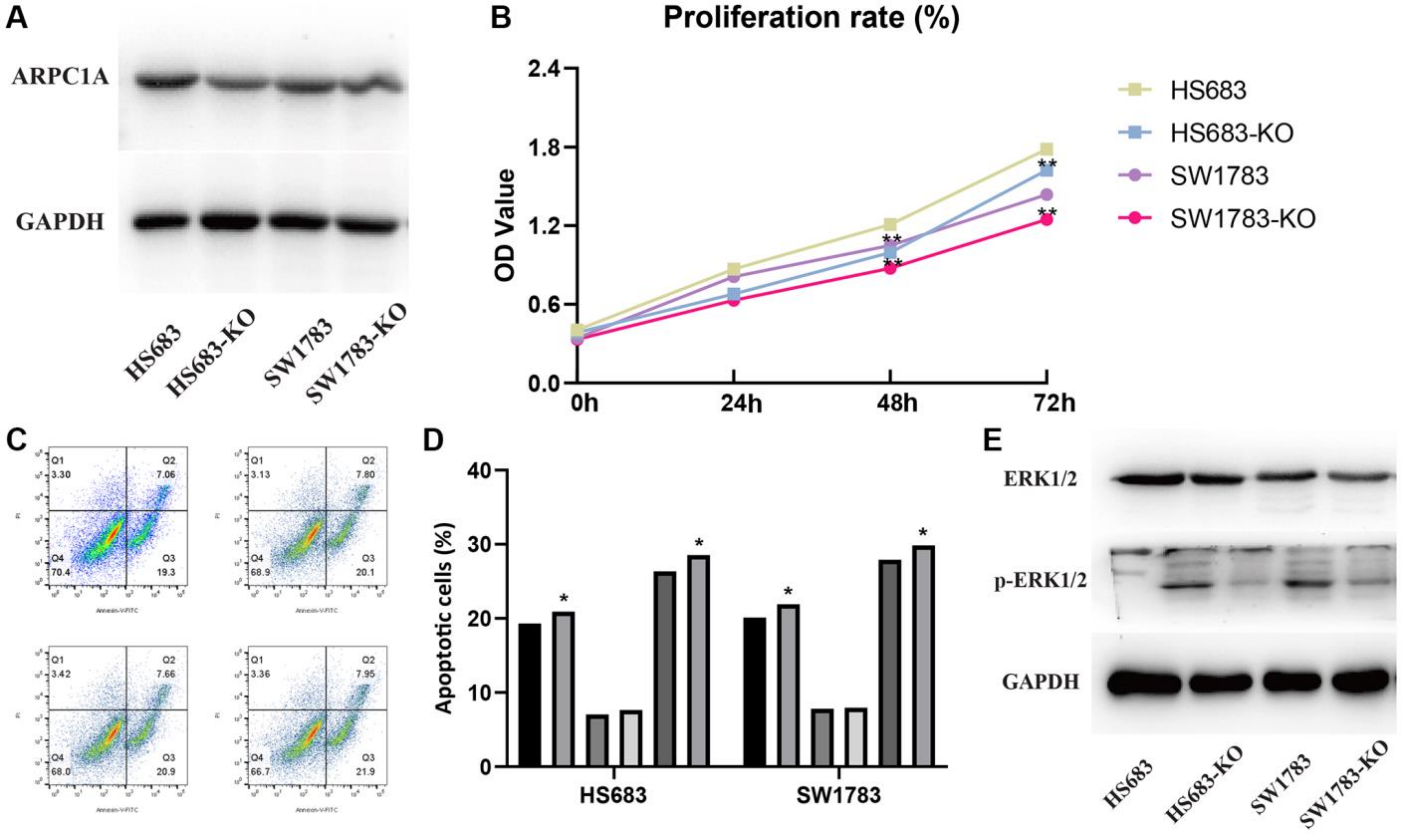
(**A**) Protein expression profiles of ARPC1A in the knockout cell lines when compared to the control group. (**B**) CCK-8 assays: cell proliferation is lesser in the ARPC1A cells in the knockout cell lines than normal cells. (**C**, **D**) Flow cytometry: the apoptosis rate is lower in the knockout cell lines than in the normal cell lines. (**E**) MAPK signaling pathway accompanied by the downregulation of p-ERK1/2 in the knockout group was more evident than in the control group.

Transfection assays in the two cell lines described the higher expression of ARPC1A (Figure 6A) when compared to knockout, whereas the CC-8 assay described the decreased proliferation ability of LGG cell lines after ARPC1A knockdown (Figure 6B). Flow cytometry analysis described that the number of apoptotic population has significantly increased upon knockdown of ARPC1A than control groups. These results typically concluded the active involvement of ARPC1A in promoting LGG proliferation and inhibiting the apoptosis of LGG cells (Figure 6C, 6D). As for vimentin analysis, we did not find a significant difference, suggesting that ARPC1A does not have a significant effect on epithelial mesenchymal transition (Supplementary Figure 5). To elucidate the involvement of the MAPK signaling pathway through ARPC1A, ERK1/2 and phosphor-ERK1/2 was verified. Upon knockdown of ARPC1A, the expression of p-ERK1/2 was inhibited (Figure 6E and Supplementary Figure 6). Therefore, we speculate that overexpression of ARPC1A in LGG promotes the progression of LGG by activating the MAPK pathway.

## DISCUSSION

LGG requires early prognostic strategies to predict overall survival. A previous study by Hongwu Li et al. described the higher FNDC4 expression as evident in the glioblastoma and its involvement in the higher proliferation rate of U87 and U251 cell lines indicating FNDC4 is associated with worse prognosis. Exogenous FNDC4 effectively impaired the M1 polarization of M0 macrophages independently without affecting M2 polarization [49]. Furthermore, the overexpression of gene signatures such as FN1, ITGA5, OSMR, and NGFR from the TCGA database is associated with poor prognosis in LGG patients [50]. A total of 1489 gene signatures have been identified which have a significant correlation with the prognosis of LGG patients. Five protective genes include *DISP2, CKMT1B, AQP7, GPR162*, and *CHGB* whereas other five risk genes include *SP1, EYA3, ZSCAN20, ITPRIPL1*, and *ZNF217* have been identified in LGG and this 10-gene signature is considered as the novel prognostic or diagnostic markers for LGG [51].

Specifically, previous studies described the ability of several molecular markers involved for predicting the prognosis of glioma and these molecular markers include MEOX2 [52, 53], PDIA5 [54], and DDX3X [55]. Other mitochondrial genes such as ABCC3, HOXA4, HOXC10, NNMT, and SCNN1B are typically increased in their expression as the grade of glioma and the higher expression of these genes have been associated with poor prognosis in LGG [56]. Other mitochondrial oxidative-stress-related genes including CTSL, TXNRD2, NUDT1, STOX1, and CYP2E1 have been reported differentially expressed with potential prediction accuracy of glioma [57]. CMC1, COX20, and UQCRB are other mitochondrial genes specifically involved in the prognosis of glioma [37]. In addition to this, the cancer-promoting mechanisms of mitochondrial genes in other tumors are gradually being elucidated. For example, the mitochondria-associated dynamin and fission proteins DRP1 and MFN1, both of which are altered in expression in hepatocellular carcinoma, lead to an increase in mitochondrial fission thereby promoting the progression of hepatocellular carcinoma [58]. In addition, E2F1 triggers the onset of apoptosis by affecting mitochondria leading to their dysfunction, endoplasmic reticulum stress and other functions [59]. In our study, ARPC1A among the mitochondrial genes exhibited a higher expression compared to the normal tissues, and low overall survival rates were observed with a higher expression of ARPC1A.

The regulatory role of MAPK in cancer is very broad, playing an important role in both proliferation, apoptosis and immune regulation [60, 61]. This signaling pathway is controlled by oxidative reduction in concert with the expression of NF-κB and Nrf2, leading to carcinogenesis [62, 63]. Lun Li et al. explored the implications of METTL1 expression in the different grades of gliomas compared to normal tissues and the study concluded that the higher expression of this gene is evident as the grades of glioma increase. Gene set enrichment analysis and other functional pathways pertinent to the METTL1 significantly related to the MAPK signaling and concluded that a higher expression of METTL1 can foster a higher proliferation rate of glioma cells implicating its prognostic relevance [64]. Another study by Tianqi Liu et al. described the efficacy of ARPC1B as a potential biomarker with therapeutic implications as it is significantly expressed in gliomas. The higher expression of ARPC1B is associated with a progressive malignancy through macrophage recruitment, EMT, invasion, or migration of glioma cells. The signaling between the macrophages and glioma cells is evident through ARPC1B by the IFNγ-IRF2-ARPC1B axis [65]. Peng Wang et al. described the prognostic relevance of SLC39A1 in gliomas as indicated by the higher SLC39A1 expression resulting in poor prognosis. SLC39A1 could foster the MMP2/MMP9 expression and glioma cell proliferation and its expression is correlated to the pathological grade, IDH mutational status, and 1p19q co-deletion [4]. However, the prognostic value and regulatory role of mitochondria-related genes in LGG are still unclear. In this study, we identified the potential prognostic value of ARPC1A in LGG through the construction of a nomogram model and found that high and low expression of ARPC1A has potential value in predicting the prognosis of patients. On the basis of the prognostic analysis, we further explored the pro-carcinogenic mechanism of this gene in LGG and found that higher expression of ARPC1A in the LGG cells and caused a mitigated apoptosis rate and a higher proliferation rate through ARPC1A-mediated MAPKinase signaling. In addition to the MAPK signaling pathway, the results of our enrichment analysis also suggest that there may be other pro-cancer mechanisms of ARPC1A in LGG, including the regulation of nucleic acid purine metabolism, the regulation of the mismatch repair process, and the impact of the cell cycle and other functions, and therefore, the regulatory mechanism of ARPC1A needs to be further explored in future studies. This gene is typically involved in the cancer-promoting activity as indicated by its enhanced expression associated with poor prognosis of patients with LGG and may be a significant independent risk factor.

However, there are some limitations to this study. All eight mitochondria-related genes analyzed in this study had prognostic value, but some failed to detect differences in expression. This may be related to the characteristics of the database samples and the sample size, and therefore further analysis by expanding the sample size is needed in the follow-up.

## CONCLUSION

This study significantly explored the prognostic relevance of mitochondria-related genes and specifically ARPC1A involvement in the progression of LGG. It can affect the proliferation of LGG by its significant association with the MAP kinase pathway. However, future studies are warranted to delineate the signaling behavior and its potential involvement in cancer progression by interacting with other cancer-promoting pathways. Nomogram studies concluded that ARPC1A could be an independent prognostic risk factor that can be considered as a promising therapeutic target.

## Supplementary Materials

Supplementary Figures

Supplementary Table 1

Supplementary Tables 2-6
