# Supplementary material for: Prognostic relevance and validation of ARPC1A in the progression of low-grade glioma
Source: Aging (Albany NY). 2024 Jun 19;16(14):11162–84. doi: 10.18632/aging.205952 (PMC11315382; doi:10.18632/aging.205952)
Supplement: Supplementary Figures [file aging-16-205952-s001.pdf]

SUPPLEMENTARY FIGURES

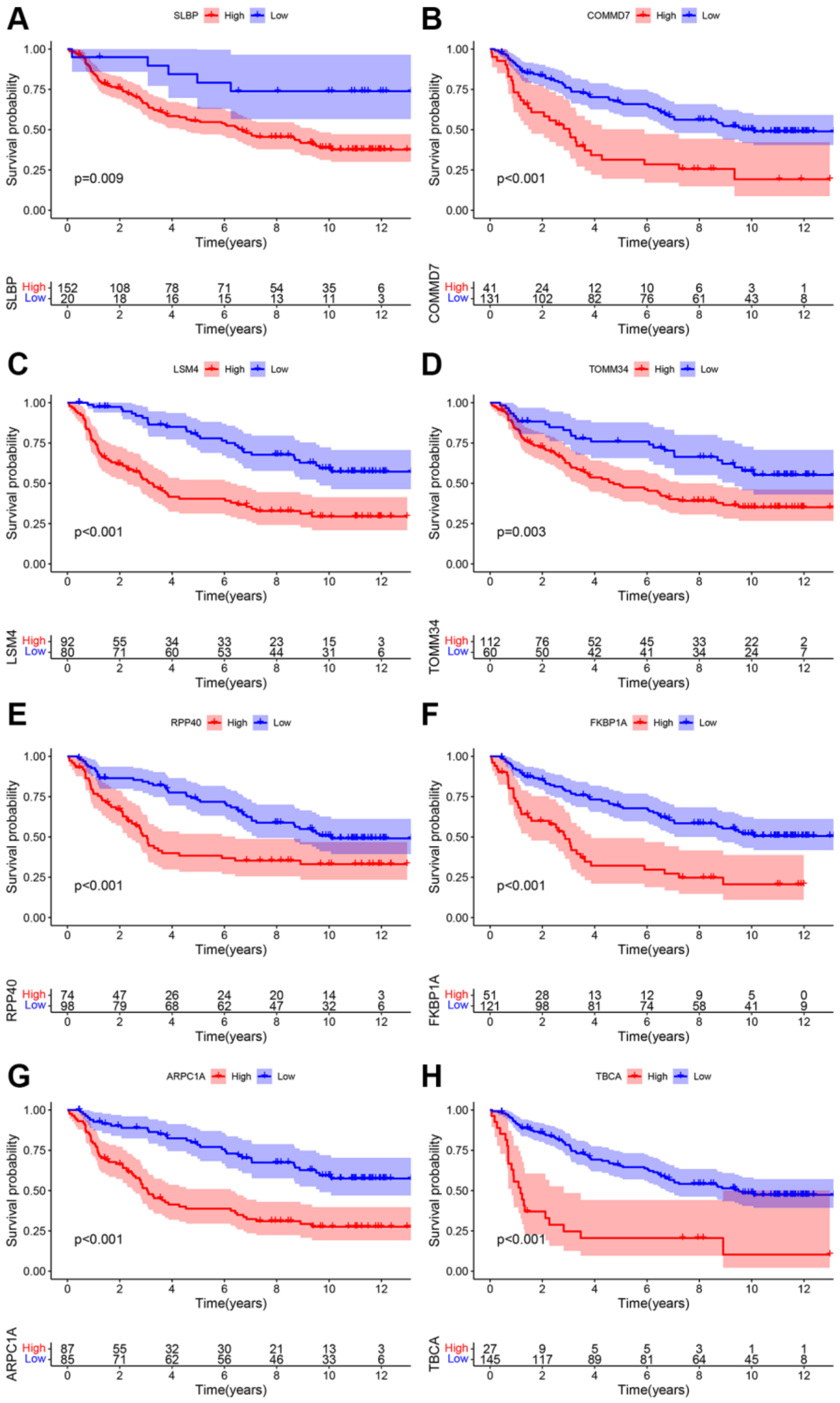

Supplementary Figure 1. (**A**–**H**) KM survival analysis curves of eight genes: SLBP, COMMD7, LSM4, TOMM34, RPP40, FKBP1A, ARPC1A, and TBCA based on the expression data and prognostic information of the CGGA database.

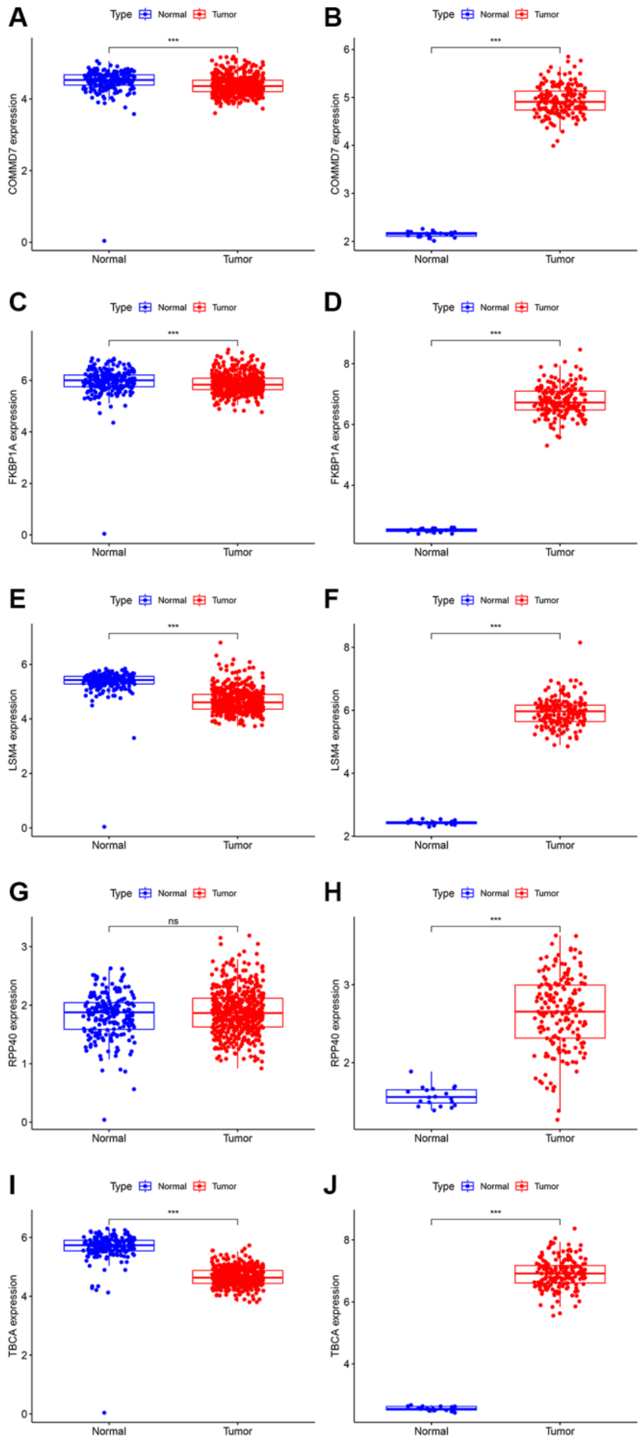

Supplementary Figure 2. (**A**, **B**) Differential expression of analysis of COMMD7 based on TCGA and CGGA expression. (**C**, **D**) Differential expression of analysis of FKBP1A based on TCGA and CGGA expression. (**E**, **F**) Differential expression of analysis of LSM4 based on TCGA and CGGA expression. (**G**, **H**) Differential expression of analysis of RPP40 based on TCGA and CGGA expression. (**I**, **J**) Differential expression of analysis of TBCA based on TCGA and CGGA expression.

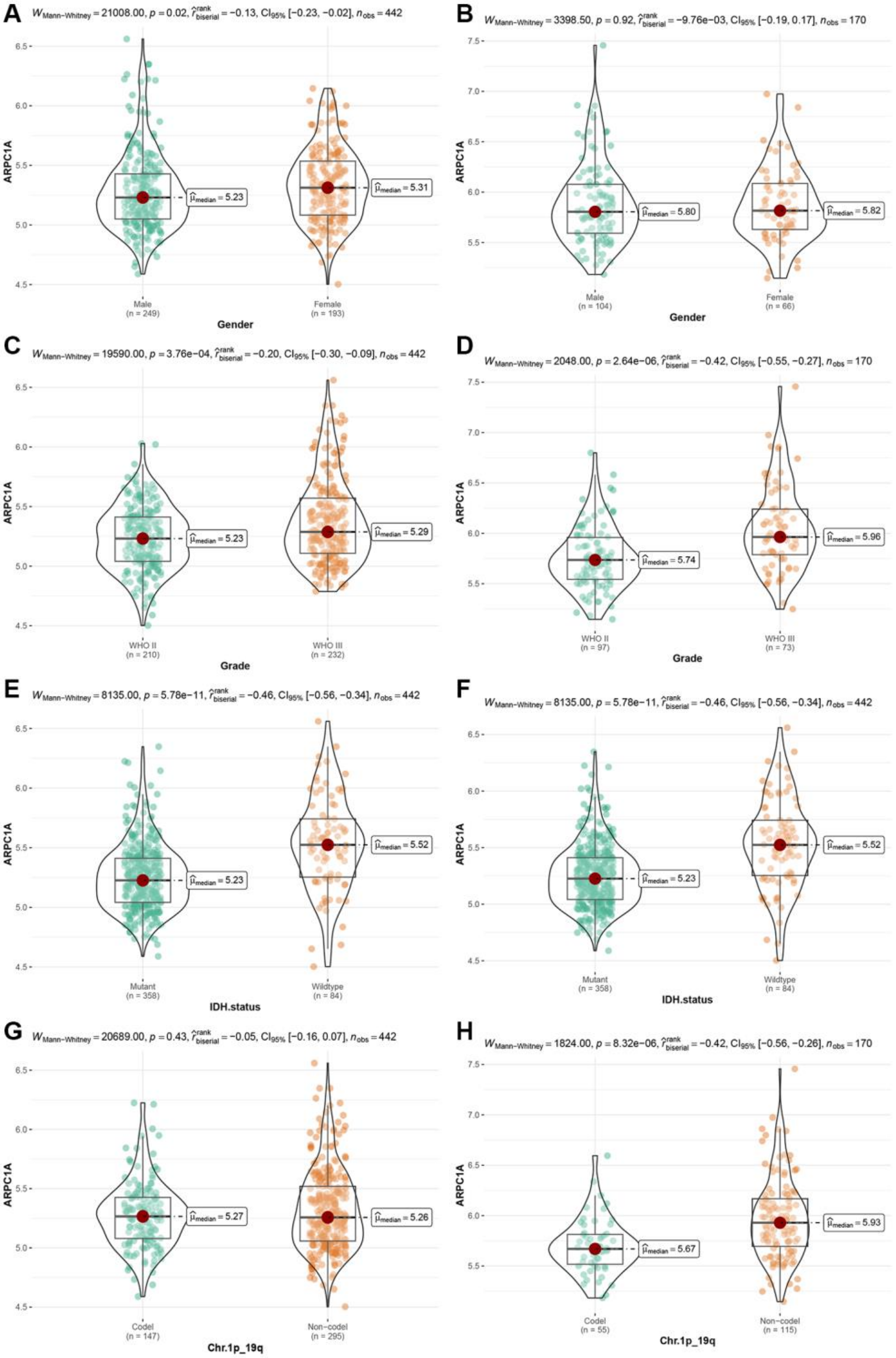

Supplementary Figure 3. Correlation analysis of ARPC1A expression with clinical risk factors for TCGA and CGGA analysis. (**A**, **B**) Gender; (**C**, **D**) Grade; (**E**, **F**) IDH mutation status; (**G**, **H**) 1p19q co-deletion state.

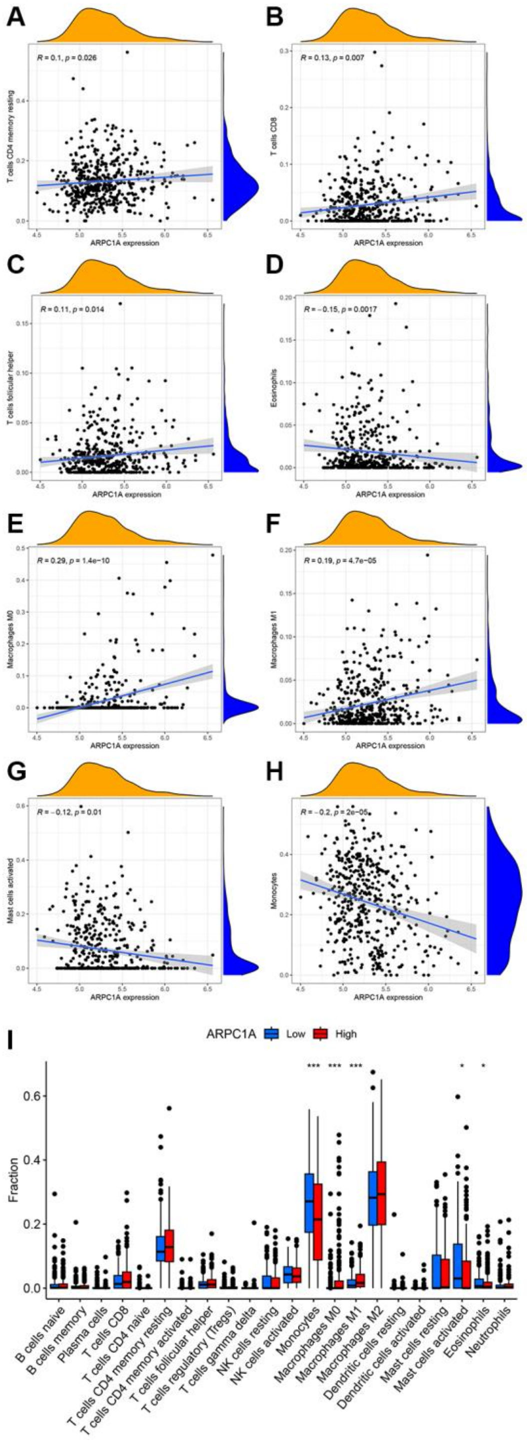

Supplementary Figure 4. Immune cell infiltration correlation analysis. (**A**) T cells CD8; (**B**) T cells CD4 memory resting; (**C**) T cells follicular helper; (**D**) Monocytes; (**E**) Macrophages M0; (**F**) Macrophages M1; (**G**) Mast cells activated; (**H**) Eosinophils; (**I**) Analysis of ARPC1A differential expression and immune cell infiltration.

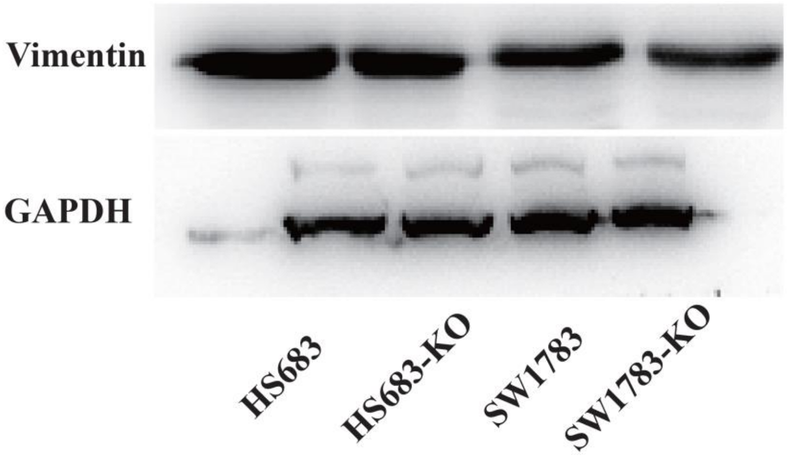

Supplementary Figure 5. Analysis of vimentin protein in four cell lines.

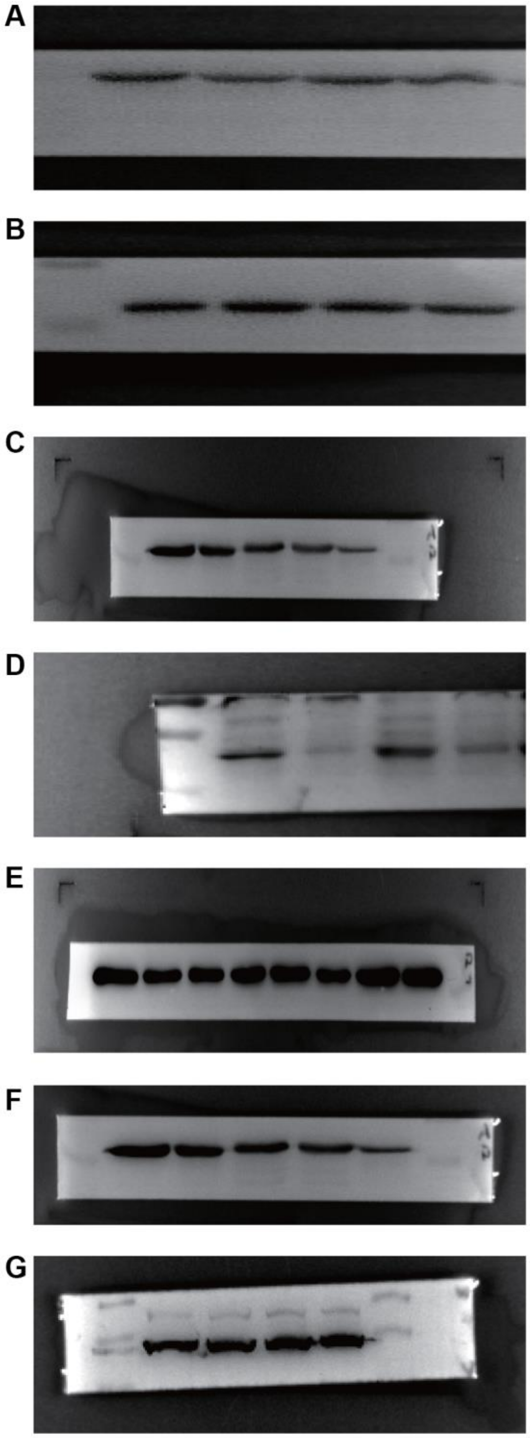

Supplementary Figure 6. (**A**–**G**) Summary of protein expression from the WB experiments involved in this study.
