# Supplementary material for: Prognostic relevance and validation of ARPC1A in the progression of low-grade glioma
Source: Aging (Albany NY). 2024 Jun 19;16(14):11162–84. doi: 10.18632/aging.205952 (PMC11315382; doi:10.18632/aging.205952)
Supplement: Supplementary Table 1 [file aging-16-205952-s002.pdf]

SUPPLEMENTARY TABLE

Supplementary Table 1. Mitochondria-related gene set.

REACTOME_MITOPHAGY
https://www.gsea-msigdb.org/gsea/msigdb/human/geneset/REACTOME_MITOPHAGY
ATG12
ATG5
CSNK2A1
CSNK2A2
CSNK2B
FUNDC1
MAP1LC3A
MAP1LC3B
MFN1
MFN2
MTERF3
PGAM5
PINK1
PRKN
RPS27A
SQSTM1
SRC
TOMM20
TOMM22
TOMM40
TOMM5
TOMM6
TOMM7
TOMM70
UBA52
UBB
UBC
ULK1
VDAC1
